# Bredemolic Acid Improves Cardiovascular Function and Attenuates Endothelial Dysfunction in Diet-Induced Prediabetes: Effects on Selected Markers

**DOI:** 10.1155/2020/1936406

**Published:** 2020-02-11

**Authors:** Akinjide Moses Akinnuga, Angezwa Siboto, Bongiwe Khumalo, Ntethelelo Hopewell Sibiya, Phikelelani Ngubane, Andile Khathi

**Affiliations:** ^1^Department of Physiology, School of Laboratory Medicine and Medical Sciences, College of Health Sciences, University of KwaZulu-Natal, Westville, Durban, South Africa; ^2^Department of Pharmacy and Pharmacology, Rhodes University, Grahamstown, South Africa

## Abstract

Prediabetes is an intermediate hyperglycaemic state which has been associated with cardiovascular dysfunction. However, cardiovascular dysfunction is not only caused by intermediate hyperglycaemia but also endothelial dysfunction, inflammation, and oxidative stress associated with prediabetes. Bredemolic acid (BA), an isomer of maslinic acid, has been reported to ameliorate the intermediate hyperglycaemia found in prediabetes; however, the effects of this triterpene on cardiovascular function have not yet been determined. Therefore, this study investigated the effects of BA on cardiovascular function in diet-induced prediabetic rats. Thirty-six male rats that weighed 150–180 g were divided into two groups, the non-prediabetic (*n* = 6) and the prediabetic groups (*n* = 30), which were fed normal diet (ND) and HFHC diet, respectively. The prediabetic rats were further subdivided into five groups (*n* = 6) and treated with either BA (80 mg/kg) or metformin (MET, 500 mg/kg) every third day for 12 weeks. After 12 weeks, blood samples and the heart were collected for biochemical analysis. The untreated prediabetic rats showed a significant increase in body mass index (BMI), waist circumference (WC), blood pressure, heart rate, lipid profile, lipid peroxidation, and inflammatory markers with significant decrease in endothelial function and antioxidant biomarkers by comparison with the non-prediabetic animals. The administration of BA significantly improved cardiovascular functions such as blood pressure, heart rate, and endothelial function. There was also a significant decrease in BMI, WC, lipid profile, lipid peroxidation, and inflammation with a concomitant increase in antioxidant capacity. BA administration improved cardiovascular function by attenuation of oxidative stress, inflammatory, and endothelial dysfunction markers.

## 1. Introduction

Type 2 diabetes mellitus (T2DM) is a heterogeneous metabolic disorder which is associated with cardiovascular diseases (CVDs), that is often preceded by the onset of prediabetes [1]. One of the identified causes of this disorder is chronic consumption of high caloric diets which are rich in carbohydrates as well as saturated and polyunsaturated fats coupled with sedentary lifestyles [2, 3]. Consequently, this leads to inefficient metabolism of carbohydrates and fats resulting in accumulation of intracellular and extracellular glucose and lipids known as glucolipotoxicity [4].

However, glucolipotoxicity is associated with insulin resistance which subsequently causes high body mass index (BMI), high waist circumference, hyperlipidaemia, oxidative stress, and release of inflammatory cytokines such as high sensitive C-reactive protein, hs-CRP, interleukin 6, IL-6, and tumour necrotic factor alpha (TNF-*α*) [3–6]. Glucolipotoxicity is also associated with endothelial dysfunction, hypertension, arteriosclerosis, coronary heart disease, and stroke [5–8]. In addition, insulin resistance is associated with decreased nitric oxide (NO) production due to inhibition of endothelial nitric oxide synthase (eNOS) via impaired phosphatidylinositol 3 kinase (PI3K)–AKT (protein kinase B) pathway [9]. The decreased NO production causes an imbalance in the vascular endothelial tone which triggers vasoconstriction followed by increased heart rate and high blood pressure [9, 10]. Prediabetes is an asymptomatic and intermediate hyperglycaemic stage that has been reported to precede the onset of cardiovascular complications observed in T2DM [8, 11, 12]. Additionally, previous studies have shown that intermediate hyperglycaemia below the level used to define diabetes mellitus is a risk factor for CVD development [13, 14].

Of note, the combination of dietary modification with pharmacotherapy is the main approach in preventing the development of CVDs in prediabetic or diabetic individuals [6, 15]. However, there has been reported low compliance to this combination therapy as most patients only observe pharmacological intervention without changing their diet [16]. This inadvertently reduces the efficacy of the pharmacological interventions [17]. Therefore, antidiabetic agents that have the ability to restore glucose homeostasis and prevent the risk of CVD development regardless of diet intervention are necessary.

Pentacyclic triterpenes such as oleanolic acid and maslinic acid are antidiabetic and antioxidant agents with proofs and literature evidence [18, 19]. More importantly, bredemolic acid (BA), an isomer of maslinic acid, has been shown in the previous study to have antidiabetic effects by reduction of blood glucose through increased expression of GLUT 4 in the skeletal muscle of prediabetic rats [20]. However, the effects of this triterpene on cardiovascular system in prediabetes have not been established. Therefore, the aim of this study was to investigate the effect of BA on selected markers of cardiovascular function in a diet-induced prediabetic rat model.

## 2. Materials and Methods

### 2.1. Animals

In this study, thirty-six male Sprague Dawley rats with body weight 150–180 g were used. The rats were obtained and bred at the Biomedical Research Unit (BRU), University of KwaZulu-Natal (UKZN). The animals were kept and maintained in standard experimental conditions at room temperature (22 ± 2°C), humidity (55 ± 5%), and 12 h day : 12 h night cycle. The animals consumed standard rat chow (Meadow Feeds, South Africa) and water *ad libitum* for 2 weeks to acclimatize before being exposed to the experimental diet (high fat high carbohydrate). The high fat high carbohydrate (HFHC) diet was composed of carbohydrates (55% kcal/g), fats (30% kcal/g), and proteins (15% kcal/g). All experimental procedures were according to the ethics and animal care guidelines of the Animal Research Ethics Committee (AREC) of UKZN, Durban, South Africa (AREC/024/018D).

### 2.2. Experimental Design

After 2 weeks of acclimatization, the animals were distributed into two main groups: the non-prediabetic control group (*n* = 6) and the prediabetic group (*n* = 30). The non-prediabetic control (NPDC) animals served as the negative control and were given normal diet (ND) and water *ad libitum* while the prediabetic animals were given HFHC diet and drinking water supplemented with fructose (15%) for 20 weeks to induce prediabetes. After 20 weeks, prediabetes was confirmed via fasting blood glucose and oral glucose tolerance test using criteria of the American Diabetes Association as described in our previous study [20].

### 2.3. Treatment of Animals

After the 20 weeks of prediabetes induction, the non-prediabetic control (Group 1) continuously fed on ND for a further 12 weeks while the prediabetic animals (*n* = 30) were divided into 5 groups (Group 2–Group 6, *n* = 6 in each group). Group 2 (PD) served as the untreated prediabetic control group and continuously consumed the HFHC diet for 12 weeks; Group 3 (ND + MET) were prediabetic animals that switched to standard rat chow and received MET for 12 weeks; Group 4 (HFHC + MET) were prediabetic animals that continuously consumed HFHC diet with MET treatment; Group 5 (ND + BA) were prediabetic animals that switched to standard rat chow and received BA for 12 weeks; Group 6 (HFHC + BA) were prediabetic animals that continuously consumed HFHC diet and received BA as treatment for 12 weeks. Treatment with either MET (500 mg/kg) or BA (80 mg/kg) was carried out twice every third day for 12 weeks. The body mass index (BMI), waist circumference (WC), blood pressure, and heart rate were assessed in all animals at week 20 and every 4 weeks (24^th^, 28^th^, and 32^nd^ week).

### 2.4. Blood Collection and Tissue Harvesting

After the 12 weeks of treatment, the animals were sacrificed. The animals were placed in a gas chamber (BRU, UKZN, South Africa) and anaesthetised with 100 mg/kg of Isofor (Safeline Pharmaceuticals Ltd, Roodeport, South Africa) for 3 minutes to collect blood samples. In an unconscious state, blood samples were collected by cardiac puncture into precooled heparinized containers. The blood samples were centrifuged (Eppendorf centrifuge 5403, Germany) at 4°C, 503 g for 15 minutes for plasma collection. The plasma were collected and stored at −80°C in a Bio Ultra freezer (Snijers Scientific, Tilburg, Holland). The hearts of all the animals were excised, rinsed with cold normal saline solution, weighed, and snapped frozen in liquid nitrogen before storage in Bio Ultra freezer at −80°C for biochemical analysis.

### 2.5. Determination of BMI and WC

The determination of BMI was measured from the ratio of the weight to the square of the length of the animals as described in the established protocol [21]. Also, the waist circumference of the animals was determined according to the previous protocol [22].

### 2.6. Determination of Blood Pressure and Heart Rate

The blood pressure and heart rate were measured as described in the established protocol [19]. Briefly, at every 4 weeks of treatment, the noninvasive MRBP IITC Model 31, Life Sciences multichannel tail cuff blood pressure system (Life Sciences, Woodland Hills, CA) was used to monitor the blood pressure and the heart rate by placing the animals in a restrainer (3″ ID (75 mm)–12″ length) while the tail of the animals is attached to the tail cuff. All the rats in the restrainer were placed in a warming chamber (IITC Model 303sc Animal Test Chamber, Life Sciences, Woodland Hills, CA) maintained at 32°C, and the blood pressure as well as the heart rate was measured by occlusion or deflation of the tail cuff which detects alteration of blood flow in the tail artery. An average of three measured sessions consisting of 15 cycles was used for statistical analysis.

### 2.7. Biochemical Analysis

The lipid profile, antioxidant, inflammatory, and endothelial markers were measured at 32^nd^ week only.

### 2.8. Lipid Profile Analysis

The plasma total cholesterol (TC), high density lipoprotein (HDL) cholesterol, and triglycerides (TG) were analysed via a Spectrostar Nano spectrophotometer (BMG Labtech, Ortenburg, LGBW Germany) by using commercial specialized kits according to the instruction from the manufacturer (Elabscience Biotechnology Co., Ltd., Houston, TX, USA). The other lipid profiles such as very low-density lipoprotein (VLDL) and low-density lipoprotein (LDL) cholesterol were calculated according to Friedewald's formula [23]. VLDL cholesterol = TG × 0.2, and LDL cholesterol = TC − (VLDL cholesterol + HDL cholesterol).

### 2.9. MDA and Antioxidant Status

The lipid peroxidation was determined by estimation of the amount of malondialdehyde (MDA) in the heart tissue homogenate according to previously described protocols [19, 24]. However, the antioxidant status of the heart homogenates was determined by using a specific ELISA kit to analyse the concentration of superoxide dismutase (SOD) and glutathione peroxidase (GPx) according to the instruction manual of the manufacturer (Elabscience Biotechnology Co., Ltd., Houston, TX, USA).

### 2.10. Determination of Endothelial Function and Inflammatory Markers

The endothelial function and inflammation were evaluated from the plasma by determination of the endothelial nitric oxide synthase (eNOS) through the commercialized ELISA kit in accordance with the manufacturer's instructions (Elabscience Biotechnology Co., Ltd., Houston, TX, USA). The inflammatory markers (TNF-*α*, IL-6, and hs-CRP) were measured in the plasma via specific ELISA kits in accordance with the manufacturer's instruction (Elabscience Biotechnology Co., Ltd., Houston, TX, USA), and the absorbance was measured via the microplate reader, Spectrostar Nano spectrophotometer (BMG Labtech, Ortenburg, LGBW, Germany).

### 2.11. Statistical Analysis

The data were presented as mean ± standard error of mean (SEM). Statistical analysis was determined by two-way analysis of variance (ANOVA) followed by the Bonferroni test as post hoc via GraphPad Prism 5 software. The level of statistical significant difference was considered at *p* < 0.05.

## 3. Results

### 3.1. Body Mass Index (BMI) and Waist Circumference (WC)

**Figure 1:**
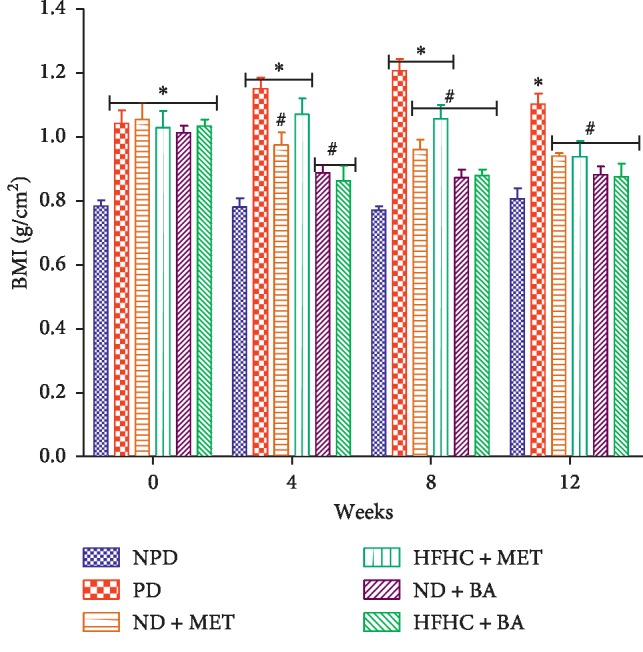
Effects of BA on BMI in non-prediabetic (NPD) and prediabetic rats with or without diet intervention. ^*∗*^*p* < 0.001 in comparison with NPD; ^#^*p* < 0.01 in comparison with PD.

**Figure 2:**
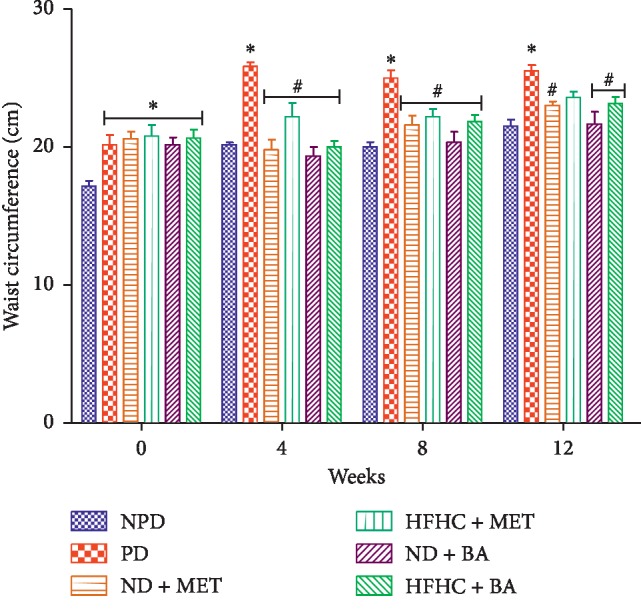
Effects of BA on waist circumference in non-prediabetic and prediabetic rats with or without diet intervention. ^*∗*^*p* < 0.001 in comparison with NPD; ^#^*p* < 0.05 in comparison with PD.

The effects of BA treatment on BMI and WC in non-prediabetic and prediabetic rats with or without diet intervention were determined as indicated in Figures 1 and 2. The BMI and WC of the untreated prediabetic (PD) rats were significantly increased by comparison with the non-prediabetic (NPD) control rats throughout the treatment period (*p* < 0.001). However, the administration of BA with or without diet intervention significantly decreased both BMI and WC when compared to the PD group as shown in Figures 1 and 2, respectively (*p* < 0.01).

### 3.2. Blood Pressure and Heart Rate

**Figure 3:**
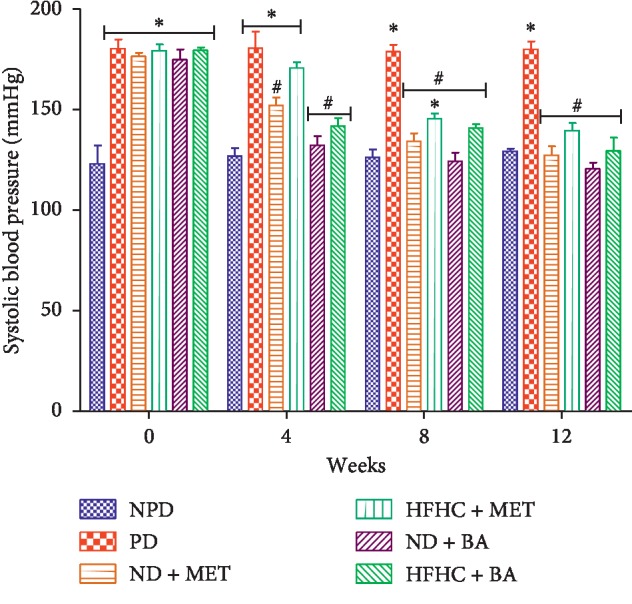
Effects of BA on systolic blood pressure in non-prediabetic and prediabetic rats with or without diet intervention. ^*∗*^*p* < 0.001 in comparison with NPD; ^#^*p* < 0.001 in comparison with PD.

**Figure 4:**
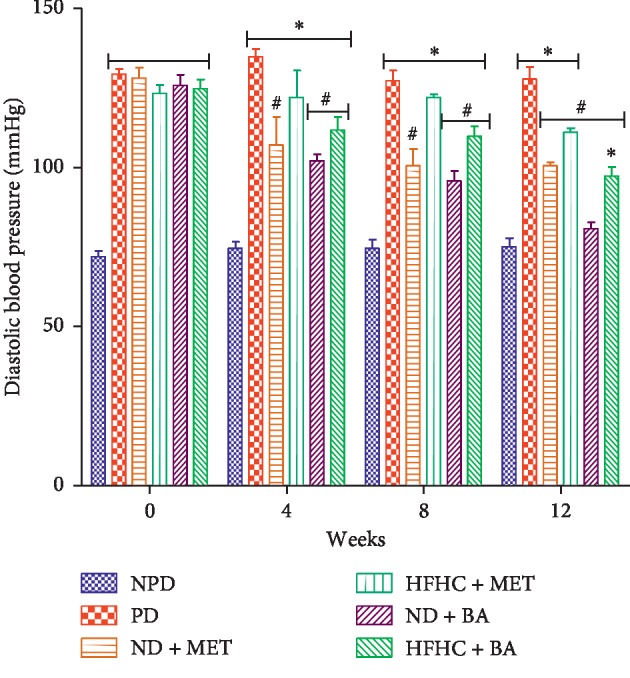
Effects of BA on diastolic blood pressure in non-prediabetic (NPD) and prediabetic rats with or without diet intervention. ^*∗*^*p* < 0.001 in comparison with NPD; ^#^*p* < 0.01 in comparison with PD.

**Figure 5:**
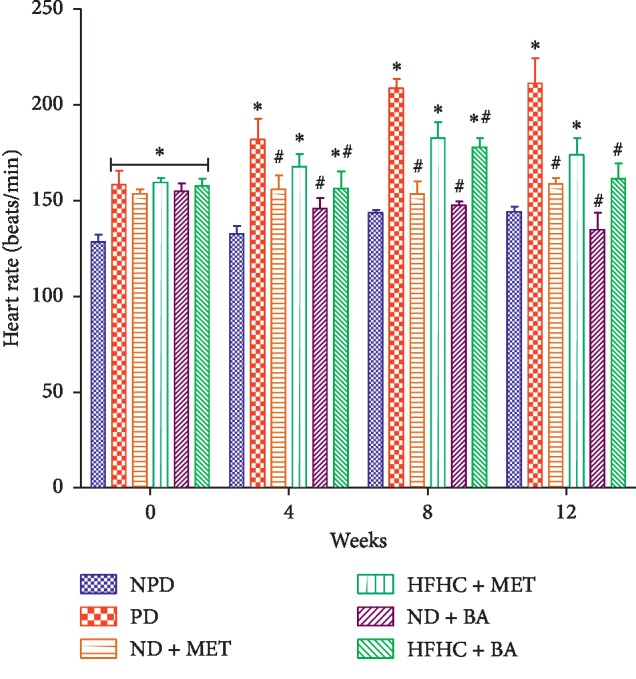
Effects of BA on heart rate in non-prediabetic (NPD) and prediabetic rats with or without diet intervention. ^*∗*^*p* < 0.001 in comparison with NPD; ^#^*p* < 0.01 in comparison with PD.

As shown in Figure 3, the systolic blood pressure of PD control rats was significantly increased throughout the treatment period when compared to the NPD control rats (*p* < 0.001). However, the systolic blood pressure of BA-treated rats with or without diet intervention significantly decreased when compared to that of PD control rats. As demonstrated in Figure 4, the diastolic blood pressure of PD control rats were significantly increased when compared to NPD control rats (*p* < 0.001). The administration of BA with or without diet intervention significantly decreased the diastolic blood pressure when compared to the PD group (*p* < 0.05). The same results were observed with the ND + MET group. A significant increase in heart rate was observed in the PD rats throughout the period of treatment when compared to the NPD control rats as indicated in Figure 5 (*p* < 0.01). However, the heart rate of BA-treated rats with or without diet intervention and MET-treated rats with diet intervention (ND + MET) were significantly lowered by comparison with the PD control rats (*p* < 0.01).

### 3.3. Lipid Profile

As shown in Table 1, the TC, TG, LDL, and VLDL of the untreated PD group significantly increased in comparison with the NPD group (*p* < 0.001). The TC and LDL of BA-treated rats with or without diet intervention were significantly decreased when compared to the PD control rats (*p* < 0.01). Similar results were obtained for the ND + MET group. Additionally, only the ND + BA and ND + MET groups had significantly lowered TG and VLDL when compared to the PD control rats (*p* < 0.05).

### 3.4. Endothelial Function Marker

**Figure 6:**
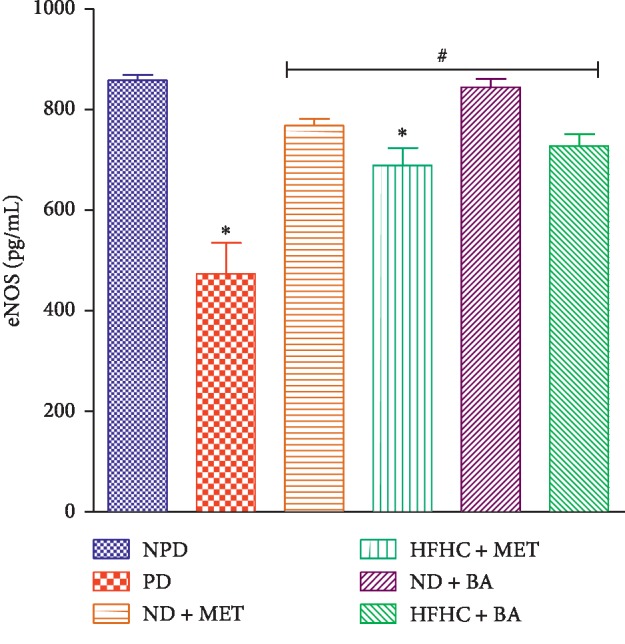
Effects of BA on eNOS concentration in non-prediabetic (NPD) and prediabetic rats. with or without diet intervention. ^*∗*^*p* < 0.001 in comparison with NPD; ^#^*p* < 0.001 in comparison with PD.

The plasma concentration of eNOS in PD control rats significantly decreased when compared to NPD control rats as indicated in Figure 6 (*p* < 0.001). However, the plasma concentration of eNOS in BA-treated rats with or without diet intervention significantly increased by comparison with the PD control rats (*p* < 0.01).

### 3.5. Lipid Peroxidation and Antioxidant Status

As indicated in Table 2, a significant increase in the heart MDA concentration was observed in the PD groups by comparison with the NPD group (*p* < 0.01). Rats treated with BA in the presence and absence of diet intervention had a significantly decreased MDA concentration by comparison with untreated PD rats. However, there was no significant difference in heart MDA concentrations in the HFHC + MET group when compared to PD control rats. The heart SOD and GPx concentration of the PD control rats significantly decreased in comparison with NPD control rats (*p* < 0.01). On the other hand, administration of BA with or without diet intervention significantly increased both SOD and GPx concentration in the heart tissue by comparison with the untreated PD group (*p* < 0.05).

### 3.6. Inflammatory Markers

As shown in Table 2, the plasma concentrations of hs-CRP, TNF-*α*, and IL-6 in the untreated PD group was significantly increased by comparison with the NPD control group (*p* < 0.001). However, the administration of BA with or without diet intervention significantly decreased the concentration of these markers by comparison with the PD group. Similar results were obtained for the ND + MET group.

## 4. Discussion

This study was designed to investigate the effects of bredemolic acid on cardiovascular function risk factors, endothelial function, oxidative stress, and proinflammatory markers in diet-induced prediabetes. High caloric diets have been implicated with prediabetes which has been associated with endothelial dysfunction, reactive oxygen species (ROS), and inflammatory cytokine production [5, 6]. Studies indicate that chronic consumption of high caloric diets promotes excess adiposity which results in high BMI, high waist circumference and hyperlipidaemia [3, 25]. These have all been identified as risk factors for developing insulin resistance, impaired glucose metabolism, and cardiovascular diseases during the prediabetic stage [26–28]. In addition, previous researchers have also shown that the risk of developing diabetes and its associated cardiovascular diseases rises as body fat, BMI, and waist circumference increase [25, 27]. Our results showed that induction of prediabetes through chronic ingestion of a high fat high carbohydrate diet significantly increased BMI and waist circumference in the untreated prediabetic rats. We suggest that the increased BMI and waist circumference can be attributed to increased caloric intake as we have reported in our previous study [20]. Conversely, the administrations of BA significantly reduced the BMI and waist circumference in BA-treated prediabetic rats with or without diet intervention. In a previous study, we reported that BA administration significantly decreased food intake through reduced plasma ghrelin concentrations and improved insulin sensitivity [20]. Therefore, in this study, we suggest that the decreased BMI and waist circumference in BA-treated prediabetic rats may be due to the decreased food intake and decreased body weight gain.

Moreover, consumption of high caloric diet has been associated with increased delivery of free fatty acid (FFA) to the liver [29]. The increased delivery of FFA leads to increased hepatic and plasma TG concentrations as well as increased export of TG as VLDL from the liver [29, 30]. The VLDL is in turn converted into atherogenic LDL with low clearance. Consequently, due to the increased conversion of TG to VLDL, HDL clearance increases and results in decreased plasma HDL concentration [31, 32].

Similarly, in this study, consumption of high caloric diet probably caused increased delivery of FFA to the liver with subsequent significant increase in plasma concentrations of TC, TG, LDL, and VLDL as well as a slight decrease in the HDL concentration in the untreated prediabetic rats. However, we suggest that even though the HDL concentration slightly decreased, the clearance of HDL as a result of increased VLDL formation remains unaffected in this study. Hence, this abnormal lipid profile showed that the risk of developing dyslipidaemia and other cardiovascular complications begins during the prediabetic stage [11]. On the other hand, the administration of BA significantly normalized the TC, TG, LDL, and VLDL levels in BA-treated prediabetic rats with or without diet intervention. In our previous study, BA was reported to inhibit caloric intake and decrease body weight gain, and this may contribute to the observed normal lipid profile in the BA-treated rats [20].

High caloric diets have also been reported to result in glucolipotoxicity which in turn triggers mitochondrial overproduction of reactive oxygen species (ROS) due to impairment of mitochondrial electron transport chain activity [4, 33]. The mitochondrial overproduction of ROS leads to oxidative stress which further leads to impaired balance between production of ROS and antioxidant enzymes [3, 34]. MDA and antioxidant enzymes (SOD and GPx) are markers for lipid peroxidation and antioxidant capacity in the cells or tissue, respectively. Indeed, in this study, MDA concentrations significantly increased while SOD and GPx concentrations significantly decreased in the hearts of untreated prediabetic rats. These results correlated with a research done by Lozano et al. [3] which showed a positive correlation between the consumption of high caloric diets and increased lipid peroxidation. On the other hand, we observed that the administration of BA significantly reduced the heart lipid peroxidation activity and significantly increased the heart antioxidant capacity of BA-treated prediabetic rats. This biological effect of BA on the oxidative stress markers correlated with the earlier reports that triterpenes are antioxidant agents which neutralize free radicals in the mitochondria by donation of electrons due to the presence of hydroxyl radical in their structures [19]. Similarly, we speculate that BA attenuated oxidative stress by neutralizing free radicals through electron donation capacity of its hydroxyl radicals and improved antioxidant activity by promotion of antioxidant enzyme production. This antioxidant property of BA has also been reported in other triterpenes such as maslinic acid, oleanolic acid, and ursolic acid [19, 35].

Studies indicate that intermediate hyperglycaemia and oxidative stress alter endothelial cell function and contribute to cardiovascular diseases during the prediabetic stage [33, 36, 37]. Intermediate hyperglycaemia has been linked to oxidative stress through the activation of protein kinase C (PKC) which in turn enhances the action of nicotinamide adenine dinucleotide phosphate (NADPH) oxidase [8, 38]. Activation of PKC alters vascular homeostasis and decreases nitric oxide (NO) production via inhibition of eNOS [1, 12]. As a result of the decreased NO production, vascular changes that result in vasoconstriction with subsequent increase in blood pressure, heart rate, and arteriosclerotic processes occur [1]. In this study, we observed that the eNOS concentration significantly decreased with concomitant increases in heart rate and systolic and diastolic blood pressure in the untreated prediabetic rats when compared to non-prediabetic control rats. The increased heart rate and systolic and diastolic blood pressure can be attributed to vasoconstriction of vascular endothelium due to decreased eNOS activity that result in decreased NO production which has been reported in prediabetes [6]. The results of this study further showed that the administration of BA significantly increased the eNOS concentration and ameliorated heart rate and systolic and diastolic blood pressure in both the presence and absence of diet intervention. In accordance to similar study, we suggest that the administration of BA which ameliorated oxidative stress contributed to the increased eNOS concentration in the BA-treated rats [39]. Increased eNOS concentration in turn leads to increase in NO production which further leads to vasodilation with subsequent significant decrease in heart rate and blood pressure when compared to untreated prediabetic rats.

Furthermore, increased blood glucose has been reported to result in formation of advanced glycation product (AGE) [40]. Formation of AGEs increases expression of adhesion molecules on vascular endothelial cells and subsequently promotes migration of monocytes to form macrophages [1, 41]. Stimulation of the monocytes by AGEs leads to low grade inflammation with increased production of cytokines (such as IL-6, TNF-*α*, and hs-CRP) [4, 41]. However, literatures have reported that increased levels of proinflammatory cytokines are associated with prediabetes [12, 14]. Similarly, in this study, the plasma concentration of IL-6, TNF-*α*, and hs-CRP significantly increased in untreated prediabetic rats. The elevated proinflammatory cytokines are inflammatory responses that alter vascular endothelium and result in endothelial dysfunction during prediabetic stage [12, 42]. Of notes, hs-CRP is not just a proinflammatory cytokine but a biomarker for injured heart caused by coronary heart disease or ischemic heart disease [43].

The observed increase in plasma hs-CRP concentrations in untreated prediabetic rats in this study indicated the risk of developing cardiovascular diseases during the prediabetic stage. These results correlated with other studies which reported that plasma hs-CRP concentration and other proinflammatory cytokines were significantly increased in prediabetic condition [44, 45]. Additionally, BA administration significantly decreased the proinflammatory cytokines such as hs-CRP, IL-6, and TNF-*α* in prediabetic rats with or without diet intervention. The decrease in the plasma proinflammatory cytokines concentration can be suggested to be due to the anti-inflammatory property that has been previously attributed to pentacyclic triterpenes [19, 35]. Pentacyclic triterpenes (such as maslinic acid and oleanolic acid) have been reported to have low pharmacokinetic activity of 3 days without any side effects [35, 46, 47]. Therefore, as a result of the low pharmacokinetic activity exhibited by the pentacyclic triterpenes, the biological effects of BA last longer and sustainably remain active than synthetic drugs. However, we suggest that the sustained biological activities of BA probably compensated for the ameliorated cardiovascular functions in the prediabetic rats even in the absence of diet intervention.

## 5. Conclusion

The findings of this study suggest that the administration of BA in both the presence and absence of diet intervention attenuated inflammation and oxidative stress, as well as improved cardiovascular and endothelial functions which are impaired in diet-induced prediabetes. More studies are, however, required to investigate the molecular mechanisms by which this triterpene exerts its biological effects.

## Figures and Tables

**Table 1:** The effects of BA on lipid profile in non-prediabetic and prediabetic rats with or without diet intervention.

Parameters	Groups					
	NPD	PD	ND + MET	HFHC + MET	ND + BA	HFHC + BA
TC (mmol/L)	2.00 ± 0.04	2.88 ± 0.03^∗∗∗^	2.06 ± 0.03^###^	2.43 ± 0.16	2.10 ± 0.09^###^	2.25 ± 0.13^##^
TG (mmol/L)	1.12 ± 0.10	1.75 ± 0.02^∗∗^	1.13 ± 0.03^##^	1.58 ± 0.22	1.18 ± 0.02^#^	1.45 ± 0.02
HDL (mmol/L)	1.11 ± 0.03	1.04 ± 0.04	1.13 ± 0.04	1.08 ± 0.09	1.16 ± 0.06	1.10 ± 0.05
LDL (mmol/L)	0.67 ± 0.04	1.49 ± 0.05^∗∗∗^	0.70 ± 0.06^###^	1.03 ± 0.05^∗##^	0.71 ± 0.07^###^	0.86 ± 0.12^###^
VLDL (mmol/L)	0.22 ± 0.02	0.35 ± 0.01^∗∗^	0.23 ± 0.01^##^	0.32 ± 0.05	0.24 ± 0.01^#^	0.29 ± 0.01

Values are presented as mean ± SEM (*n* = 6). ^*∗*^*p* < 0.05, ^*∗∗*^*p* < 0.01, ^*∗∗∗*^*p* < 0.001 (vs. NPD). ^#^*p* < 0.05, ^##^*p* < 0.01, ^###^*p* < 0.001 (vs. PD).

**Table 2:** The effects of BA on oxidative stress and inflammatory biomarkers in non-prediabetic and prediabetic rats with or without diet intervention.

Parameters	Groups					
	NPD	PD	ND + MET	HFHC + MET	ND + BA	HFHC + BA
MDA (nmol/g protein)	4.35 ± 0.16	5.78 ± 0.43^∗∗^	4.62 ± 0.09^#^	5.11 ± 0.24	4.14 ± 0.20^###^	4.53 ± 0.12^#^
SOD (ng/mL)	7.00 ± 0.90	1.78 ± 0.23^∗∗^	6.74 ± 0.66^##^	6.11 ± 0.88^#^	11.43 ± 1.14^∗###^	10.56 ± 0.90^∗###^
GPx (pg/mL)	847.52 ± 53.56	245.43 ± 12.29^∗∗∗^	989.72 ± 129.55^###^	517.99 ± 78.53^∗^	1001.20 ± 62.37^###^	669.51 ± 40.59^##^
hs-CRP (ng/mL)	1.35 ± 0.06	2.22 ± 0.01^∗∗∗^	1.53 ± 0.16^###^	1.74 ± 0.15	1.71 ± 0.07^#^	1.73 ± 0.07^#^
TNF-*α* (pg/mL)	948.42 ± 30.79	1296.97 ± 7.98^∗∗∗^	1005.49 ± 19.17^##^	1108 ± 96.11	945.63 ± 13.49^###^	1011.33 ± 17.83^##^
IL-6 (pg/mL)	22.20 ± 2.71	37.13 ± 1.14^∗∗∗^	30.02 ± 1.30	33.95 ± 2.10^∗∗^	23.46 ± 2.50^##^	24.06 ± 1.71^##^

Values are presented as mean ± SEM (*n* = 6). ^*∗*^*p* < 0.05, ^*∗∗*^*p* < 0.01, ^*∗∗∗*^*p* < 0.001 (vs. NPD). ^#^*p* < 0.05, ^##^*p* < 0.01, ^###^*p* < 0.001 (vs. PD).

## Data Availability

The data used in this study to support our findings are available upon request from the corresponding author. However, the data on body weight, food intake, as well as fasting blood glucose and oral glucose tolerance test for confirmation of prediabetes are reported in our previous study.
